# Improving the performance of HPLC–MS using radial flow splitting columns: the analysis of caffeine in energy drinks

**DOI:** 10.1007/s00216-025-05915-y

**Published:** 2025-06-02

**Authors:** Stephen Copsey, Brandon Menacho, Leen Rafoo, Christopher E. Karlsen, Jake A. Cravino, Arianne Soliven, Feng Li, R. Andrew Shalliker, Peter J. Mahon

**Affiliations:** 1 School of Science, Computing and Emerging Technology, Swinburne University of Technology, Hawthorn, VIC Australia; 2 School of Science, Western Sydney University, North Parramatta, NSW Australia; 3 Australian Centre for Research On Separation Science (ACROSS), Western Sydney University, Penrith, Australia

**Keywords:** Radial flow splitting, Liquid chromatography, Mass spectrometry, Caffeine, Energy drinks, Throughput

## Abstract

The management of the mobile phase at the interface between high-performance liquid chromatography (HPLC) and mass spectrometry (MS) has always been important. Due to the necessity to operate the MS under a state of high vacuum, the removal of the mobile phase from the HPLC has always presented as a problem. Hence, the chromatographic separation is limited in flow rate by the capacity of the MS to remove the subsequent mobile phase vapor prior to solute transport to the detection source. Although the efficiency of solvent removal at the interface between HPLC and MS has improved over the decades, there is an increasing requirement for HPLC columns to operate at higher linear flow velocities. If this is to be done without the use of narrow bore or capillary columns, this necessitates the use of higher volumetric flow rates. It is this disparity that continues to make the marriage between HPLC and MS problematic. Herein, we have tested the performance of HPLC with MS detection by incorporating recently developed radial flow stream (RFS) splitting end fittings to standard HPLC columns. These RFS end fittings enable the total flow of the mobile phase to be split so that only the central portion is directed to the MS, which actually increases the efficiency in the separation step due to the flow profile in the column. In essence, the incorporation of the RFS end fittings enabled a threefold increase in analytical throughput compared to standard HPLC columns without any loss in quantitative metrics, and other key findings are also presented.

**Graphical Abstract:**

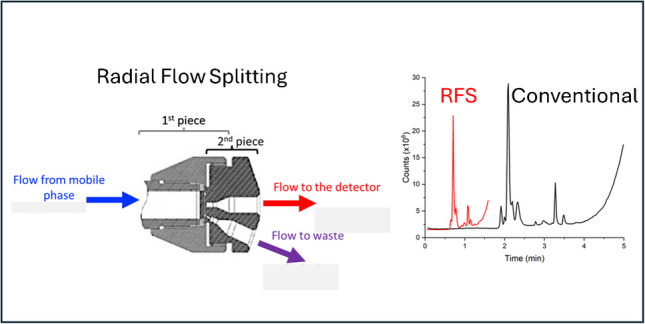

## Introduction

Since the origins of the hyphenation of HPLC with MS, there has always been an issue with the management of the mobile phase flow into the mass spectrometer [1]. In the early days, columns operated either at flow rates well below their optimum according to a height equivalent to a theoretical plate HETP plot [2, 3], or with relatively acute post-column flow stream splitting [2, 4]. In either scenario, the separation performance is sacrificed for the benefit of the coupling between the HPLC and MS [5]. Fast forward to the modern situation. Now, the MS can manage a much higher inlet flow, as the source (usually electrospray) is often heated, and there is a much more efficient curtain flow of gas that serves to vaporize the mobile phase exiting the column prior to entry into the MS. Typically nowadays, depending on the nature of the mobile phase, flow rates anywhere between 0.7 and 1.0 mL/min can be accommodated, even as high as 1.5 mL/min depending on the duration of the assay or series of assays [4, 6, 7], but lower flow rates still yield higher sensitivities [1]. In many instances, such flow rates are quite compatible with the optimum in the HPLC system, and hence it appears the problem of the interface between the HPLC and the MS has been solved, even allowing for the separation using 4.6 mm i.d. columns. If highly aqueous solvents are required and flooding of the source occurs when using 4.6 mm i.d. columns, the inner diameter of the column can be reduced to either 3.0 mm or 2.1 mm [8, 9], which thus serves the purpose of optimal flow in the HPLC and kinder solvent delivery to the MS [10, 11].

It appears then that there is nothing to be gained by further advancements in the management of fluid flow between the HPLC and the MS. However, complacency in design and uptake in new technologies will ultimately lead to a plateau in performance when gains in HPLC enable even faster flows. Such situations currently are developing. Particles are decreasing in size [12], systems can accommodate higher pressures [13], and now these types of columns can maintain high efficiency at flow rates well beyond the capacity of the MS to process the solvent flow prior to entry (an example of which is shown in reference [14]). We also have monolithic columns, which offer a robust separation medium that can tolerate high flow rates with respect to separation efficiency. Hence, considering the ever-increasing demand in a commercial sector to undertake more and more analysis, there still remains the need to go faster. Therefore, work is still required at the interface.

New advancements in column technology, specifically in the design of the column outlet, have enabled very efficient separations to be undertaken. Radial flow stream (RFS) splitting has shown benefits with respect to removing radial heterogeneous flow effects due to the wall effect and radial thermal temperature variations associated with viscous frictional heating that occurs when a column is operated at high flow velocity and/or high pressure [15]. These radial flow stream splitting end fittings have been described in previous publications, and detailed design benefits need not be reiterated here. However, these prior publications predominantly used UV–Visible detectors, which measure the concentration of the solute, whereas a mass spectrometer detects the amount of solute. This distinction, as related to the RFS fitting, requires further investigation.

Suffice to say, it is possible to envisage three distinct advantages of these radial flow stream splitting end fittings as they relate to the coupling between the HPLC and the MS: [1] The column operates at higher efficiency than a conventional column, and the gain in efficiency compared to a conventional column increases as the flow rate increases. Hence, they maintain better separation performance at higher flow rates. This improvement to column performance (corresponding to narrower peaks) improves peak capacity, and thus in areas of the separation in which selectivity and resolution are poor and fail to resolve the compounds of interest, the RFS technology is helpful to further resolve the compounds—thus reducing the matrix effect of closely eluting species. [2] There is a reduction in operational pressure when using the end fittings described in [15]. The multiport outlet allows for a reduction (commonly 20% [15]) of the pressure compared to the conventional column. Couple that with the gains in efficiency seen by radial flow stream splitting, and you now have a column that can run substantially faster than a conventional column but with similar separation power or better. [3] The mobile phase exiting the column does so through two separated flow channels—one is located in the radial central region of the column and the other is located on the peripheral of the column radius—near the wall of the column. These two flow regions can be managed separately, and ideally, the flow from the radial central region of the column is sent to the detection source since this part of the flow profile yields the best efficiency and sensitivity. Typically, around 30 to 40% of the flow should exit the radial central port for the most efficient separation efficiency but less can be tolerated if required for higher throughput to the MS. Subsequently, these three key advantages of radial flow stream splitting produce a situation whereby it is not uncommon to be able to run the HPLC separation three times faster with radial flow stream splitting into the MS than when a conventional column is employed (and this is the case demonstrated in the present work). The gains in separation performance obtained when radial flow stream splitting is employed are more or less consistent across column formats that are typical of being coupled to an MS (i.e., 2.1 to 10 mm). In the present study, we show the benefits of the radial flow stream splitting outlet column to analyses undertaken using MS detection.

In this work, the radial flow stream splitting end fitting is attached to a monolithic column, which maintains high efficiency even at flow rates around 2.5 mL/min [15]. Using a conventional monolithic column at these high flow rates is entirely incompatible with the MS, but with radial flow stream splitting, a simple adjustment of the amount of solvent exiting the column through the radial central exit port enables fast, efficient separations to be achieved. Our study demonstrates the advantages of RFS in the analysis of caffeine in energy drinks.

## Experimental

### Chemicals and reagents

Reagent grade caffeine (99%) was purchased from Sigma-Aldrich. LCMS Chromasolv grade acetonitrile was obtained from Chemsupply Australia, and Milli-Q water was obtained in-house from a Millipore system. A selection of caffeinated beverages—Pepsi, Coca-Cola, Monster Ultra Gold Zero, V Blue, Original Mother, V Green Apple Lemonade, Purple Red Bull, and Monster Pipeline Punch—was purchased from a local store, and as they are commercial samples, they have been deidentified. The beverages were degassed to remove carbon dioxide and then diluted 1 in 40 with Milli-Q water for analysis.

### HPLC column

A conventional Chromolith® HighResolution® HPLC monolithic column (C18) 100 × 4.6 mm was purchased from Merck, Darmstadt, Germany. Radial flow stream splitting end fittings were supplied by Chromaspeed Pty Ltd (Tonsley, South Australia), and these were fitted to the monolith for the purpose of this study. Once the end fitting was fitted to the column, the column could be operated in either a conventional mode of operation or in a radial flow stream splitting mode of operation. For operation in conventional mode, the peripheral port can be simply closed using an endcap. This is a distinct design feature of the Chromaspeed fitting compared to earlier designs that could only be operated in radial flow stream splitting mode [16].

### Instrumentation

Analyses were performed using an Agilent 1290 Infinity II UPLC with an Agilent 6545 Q-TOF HRMS detector that was controlled by MassHunter Data Acquisition Version 11.0 software. Agilent Jet Stream (AJS) ESI mode was used and the source parameters were gas (N_2_) temperature 320 °C; gas flow 8 L/min; nebulizer pressure 35 psi; sheath gas (N_2_) temperature 350 °C; sheath gas flow 11 L/min; VCap +3.5 kV; nozzle voltage 1000 V; and fragmentor voltage 175 V. The m/z range was set to 100–3000 with a scan rate of 1 spectra/sec. An autosampler with 2 μL injections was used and detection was in positive ion mode. Caffeine quantitation was based on 8 standard calibration curves with *R*^2^ > 0.997.

### Column performance comparison

To compare the performance of the column between the RFS and conventional modes, a column efficiency experiment was undertaken in the form of a HETP plot. For this portion of the work, the column was tested on an Agilent 1290 UHPLC system with a UV–visible detector set at 254 nm, sampling at a rate of 80 Hz. These tests were at varying flow rates using isocratic elution. The mobile phase consisted of 70% methanol and 30% water with no other mobile phase additives, and all injection volumes were 2 µL. Where the column was tested in RFS mode, ~ 35% of the flow was directed out of the central port. The retention factor of the naphthalene was ~ 1.7 with the void time being estimated by the baseline disturbance method.

The HETP performance data was calculated using the 5 sigma (5σ) report template available in the Agilent OpenLab software. Pressure was measured using the pressure readout onboard the OpenLab software and was recorded directly with no correction for extra column pressures.

### Separation conditions

For the analysis of the caffeinated beverages, a binary mobile phase was used, with solvent A being Milli-Q water and solvent B being acetonitrile. The gradient program is described in Table 1, with a flow rate of 0.7 mL/min for conventional mode and 2.1 mL/min for RFS. All analyses were performed as a batch, with an injector delay time of 2 min between the end of a run and the next injection, which contributes to the equilibration time for each run.

**Table 1:** Mobile phase gradient programs

%B	Time—RFS (min)	Time—conventional (min)
10%	Initial	Initial
60%	0.95	3.00
10%	0.99	3.10
Run time	1.60	5.00
Flow rate (mL/min)	2.1	0.7

## Results and discussion

**Fig. 1:**
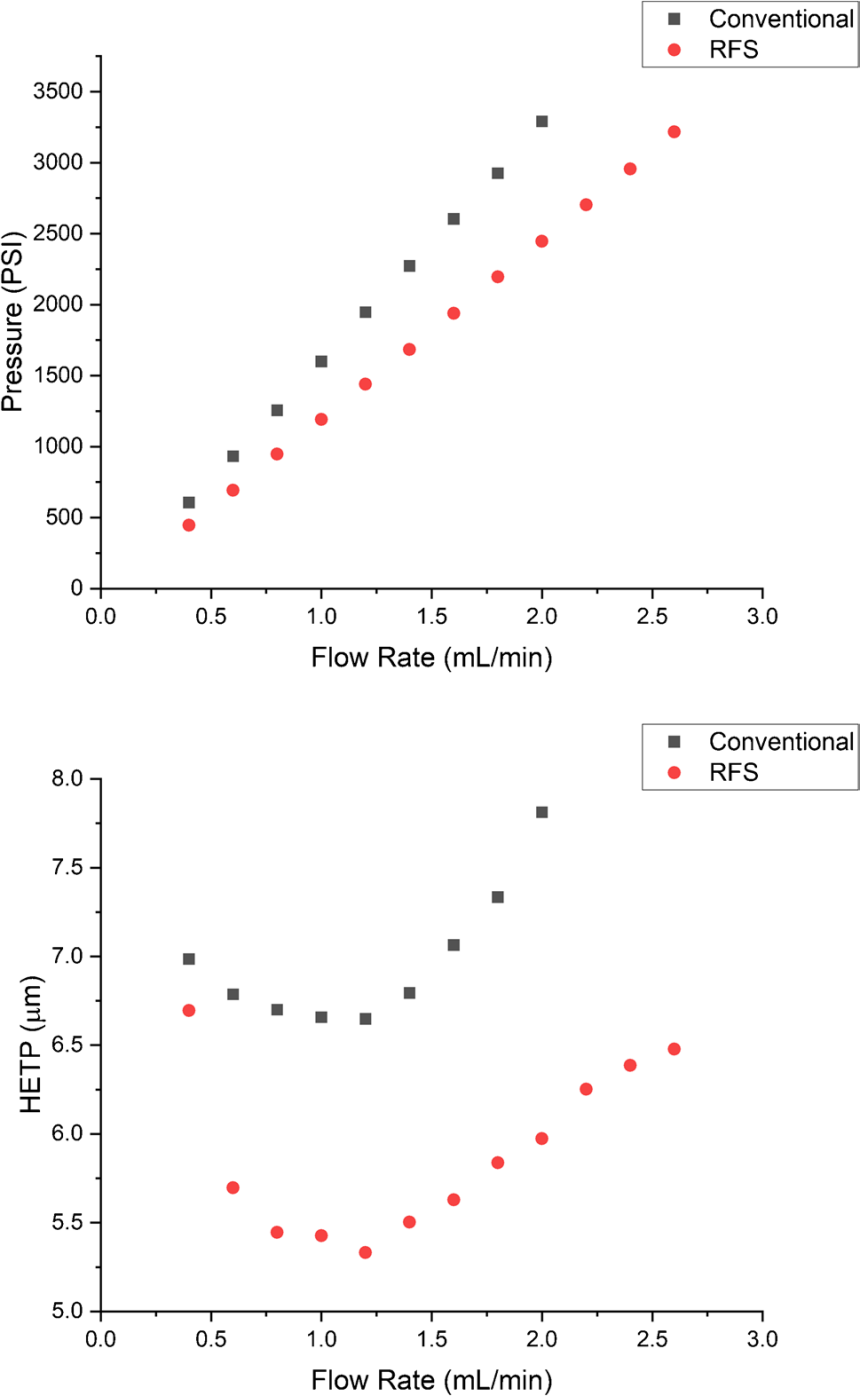
Pressure (upper) and HETP (lower) plots where the RFS mode has a 1:2 split flow between the central and peripheral ports. The mobile phase was 70:30 methanol:water. The column was tested on an Agilent 1290 with a UV–visible detector set at 254 nm, using a 2 μL injection of a 0.8 mg/mL solution of naphthalene in methanol

A comparison of the retention characteristics for the pressure and HETP is included in Fig. 1. As has been described previously [15], it is apparent in the upper plot of Fig. 1 that RFS mode has a decreased pressure of 25% compared to the conventional mode, and this can allow greater flow rates in RFS mode if pressure limits are of concern. The HETP comparison is shown in the lower plot of Fig. 1, and it is clear that the efficiency of RFS mode is greater due to only the central section of the flow stream being sampled. It follows that a greater flow rate can be used in RFS mode and still maintain a higher efficiency compared to the conventional mode when using the optimum flow rate. In this particular instance, the number of theoretical plates at 0.7 mL/min for the conventional mode of operation was still less than the number of theoretical plates in the RFS mode of operation at 2.1 mL/min (lower Fig. 1).

**Fig. 2:**
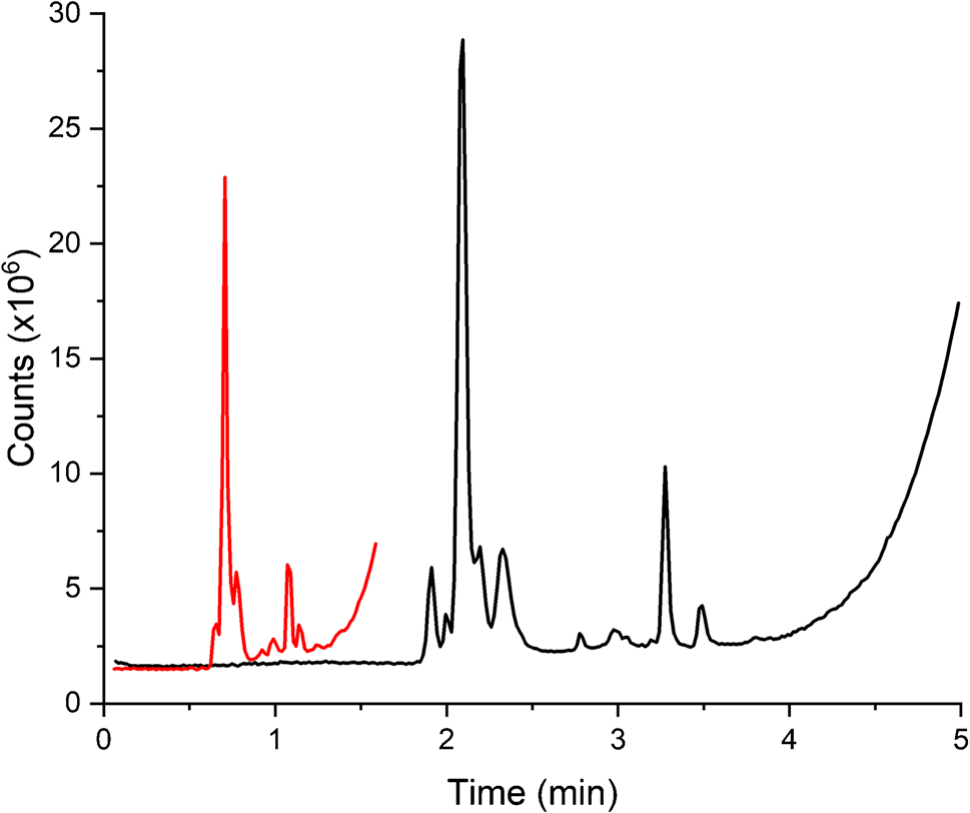
TIC of CB8 in RFS (red) and conventional (black) flow modes. The mobile phase consisted of 100% milli-Q water (solvent A) and HPLC grade acetonitrile (solvent B). The gradient program is described in Table 1 with a flow rate of 0.7 mL/min for conventional mode and 2.1 mL/min for RFS. All analyses were performed as a batch with an injector delay time of 2 min

Aside from caffeine, the energy drinks contain a number of other components. While not a complex separation problem, the number of components and their variation in hydrophobicity necessitated the use of gradient elution, at least for applications utilizing the conventional mode of operation since the efficiency was lower than in the RFS mode. The detailed gradient elution conditions utilized herein were set according to the ability of the conventional column to operate at the required level of separation performance, with additional consideration to the flow capacity of the MS. As such, the flow rate was set to 0.7 mL/min so as to not flood the MS ion source due to the highly aqueous nature of the mobile phase. The chosen gradient conditions gave the necessary separation performance. A typical chromatogram (total ion count) of an energy drink separated using the conventional monolithic column is shown in Fig. 2 (black line).

When the column was operated in radial flow stream splitting mode, the flow segmentation ratio (i.e., the proportion of flow exiting the radial central exit port to that of the flow exiting the peripheral port) was set to 1:2. That is, 33% of the flow left the column through the radial central exit port, and this was directed to the MS. Hence, the mobile phase flow rate was increased from 0.7 to 2.1 mL/min (with a corresponding adjustment of the gradient time). As a result, the separation was completed in about a third of the time compared to the separation in conventional mode. The TIC chromatograms of the same energy drink are shown in Fig. 2, and even though the separation on the radial flow stream splitting column was undertaken at a higher flow rate, the resolution was at least comparable to the resolution obtained in the conventional mode.

**Fig. 3:**
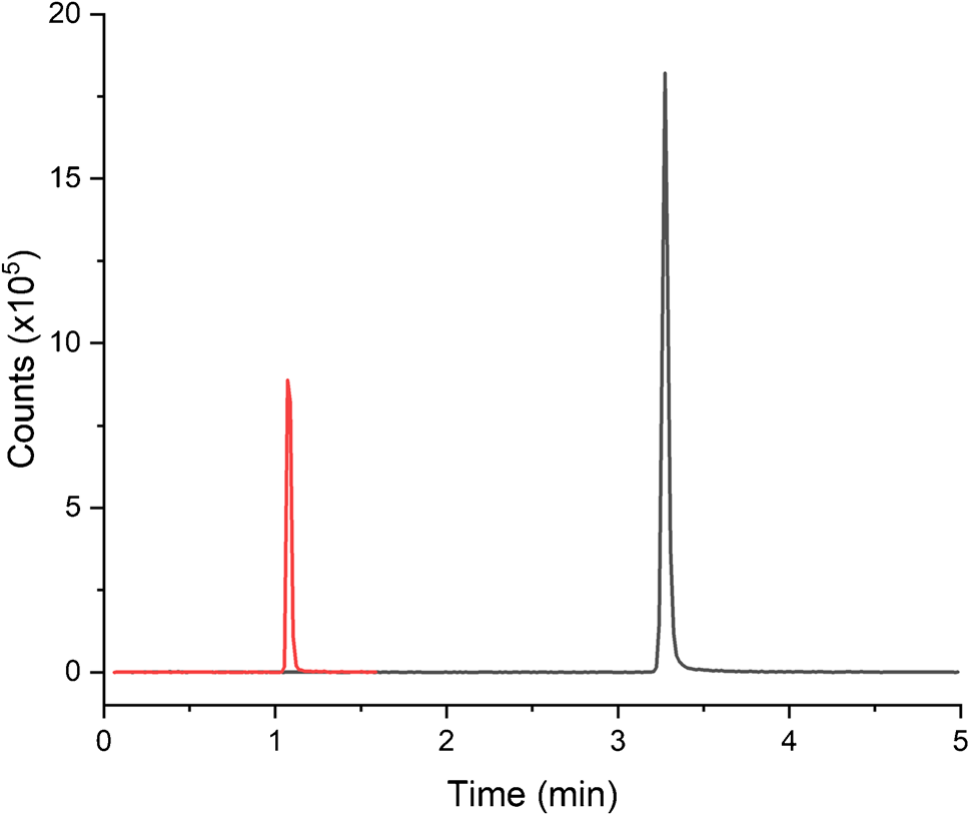
EIC of CB8 in RFS (red) and conventional (black) flow modes at 195.0882 m/z. The mobile phase consisted of 100% milli-Q water (solvent A) and HPLC grade acetonitrile (solvent B). The gradient program is described in Table 1, with a flow rate of 0.7 mL/min for the conventional mode and 2.1 mL/min for RFS. All analyses were performed as a batch, with an injector delay time of 2 min

Detection using a mass spectrometer enables EIC chromatograms to be obtained, and Fig. 3 is obtained from Fig. 2 based on the 195.0882 m/z ion, which corresponds to the [M + H]^+^ ion for caffeine. It is apparent that the peak height is approximately 1:2, matching the flow rate ratio of 1:2 for the two flow modes. In addition, the RFS peak is also much narrower than the corresponding conventional peak. The disadvantage of this narrow peak is that the acquisition rate of the mass spectrometer (1 spectra/second) means that the chromatographic peak is described by only 5 or 6 data points. However, it can be seen in Table 2 that analysis results for the seven caffeinated beverages are very similar and align closely with the expected values. This corroborates a recent finding that chromatographic peaks retain both accuracy and precision, where the peaks are described by as few as seven data points [17].

**Table 2:** Caffeine concentrations obtained from EIC chromatograms

Sample	RFS (mg/100 mL)	Conventional (mg/100 mL)	Expected (mg/100 mL)
CB1	11.2	11.9	10.7
CB2	10.3	10.7	9.6
CB3	29.7	30.7	31.7
CB4	31.7	31.0	31
CB5	31.4	30.9	31.9
CB6	30.4	30.1	31
CB7	31.2	30.2	31
CB8	32.6	31.2	32.1

**Fig. 4:**
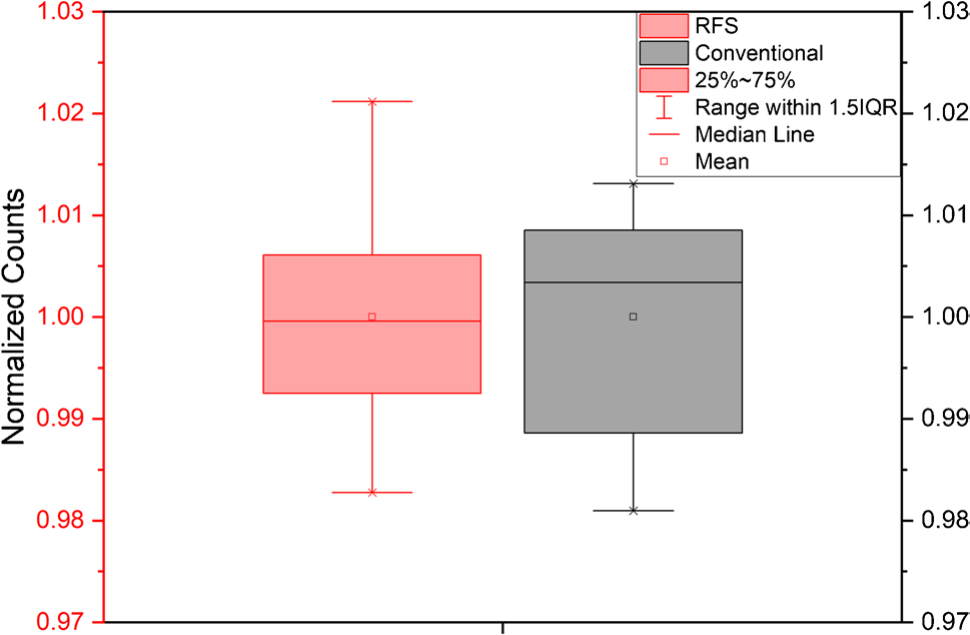
Box plot comparison for the repeatability of 10 measurements of the CB8 sample

A repeatability comparison was performed by injecting the CB8 sample ten times for each of the flow modes. Overall, the %RSD values were 1.12% for RFS mode and 1.15% for the conventional mode, which is reasonable for LCMS but also marginally surprising given that the signal magnitude is less and there are fewer data points that form the peak for RFS mode. Figure 4 provides further analysis using a box plot that shows for RFS mode, the mean and median values are almost identical, with the conventional mode showing a negative skewness. It is apparent that the results from RFS mode are not significantly different compared to the conventional mode. The decrease in signal intensity in RFS mode is the greatest disadvantage due to flow splitting, which could affect the detection limit, but it is not a problem for this analysis.

**Fig. 5:**
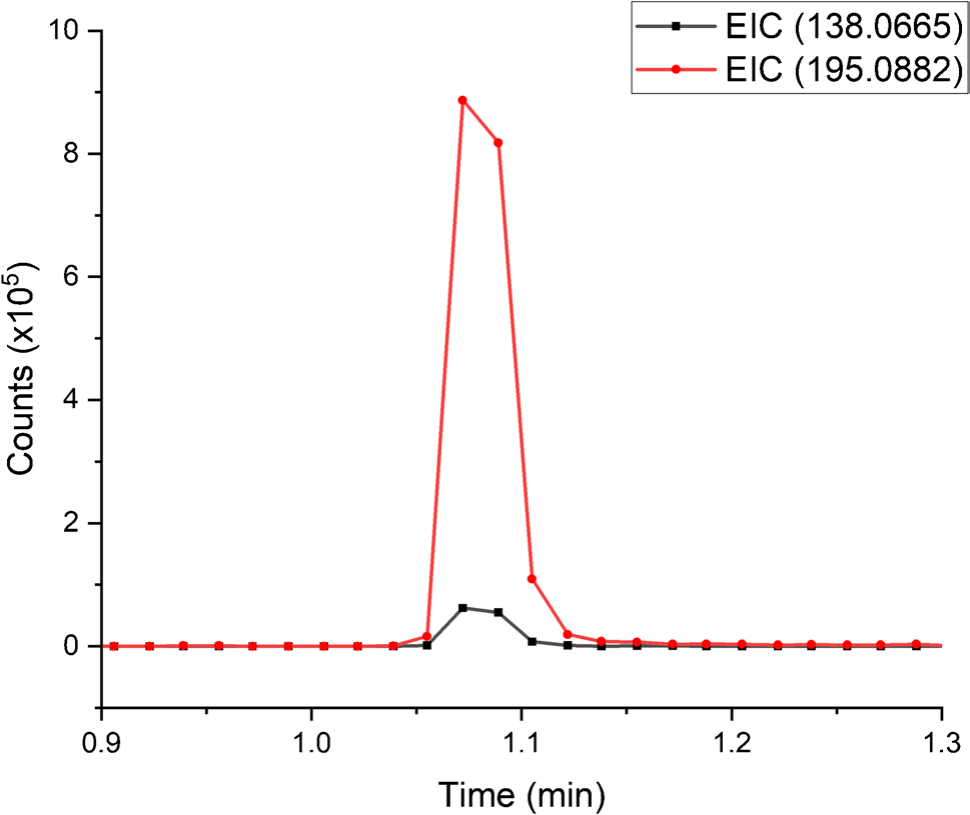
EIC of trigonelline (138.0665 m/z) overlaying the EIC for caffeine (195.0882 m/z) for the CB8 sample in RFS mode. The mobile phase consisted of 100% milli-Q water (solvent A) and HPLC grade acetonitrile (solvent B). The gradient program is described in Table 1 with a flow rate of 2.1 mL/min for RFS. All analyses were performed as a batch with an injector delay time of 2 min

Table 3 lists other components that can be identified in the caffeinated beverages based on their exact masses. The composition of energy drinks has been widely described [18] and is not a priority of our study, but there are a few noteworthy observations that can be reported. As the analyses were performed in positive mode, there are many components that have been detected as sodiated ions that have formed due to sodium salts, such as sodium citrate, being in the ingredients. One component to highlight is trigonelline because it coelutes with caffeine under the LC conditions that have been used, with an example given in Fig. 5 for CB8 measured in RFS mode. Trigonelline is found in coffee extracts [19] and if this is the source material for the caffeine in the energy drinks, then it could be difficult to resolve chromatographically. This could lead to an overestimation of the caffeine concentration when using non-specific detectors such as UV–visible or fluorescence [20].

**Table 3:** Identified compounds

Chemical formula	Detected ion (m/z) Exact mass (Da)	Identity	RFS retention time (min)	Conventional retention time (min)	Sample
C_2_H_7_NO_3_S	148.0045 125.0151	Taurine	0.693–0.705	2.051–2.079	CB3, CB5, CB6, CB7, CB8
C_12_H_22_O_11_	365.1068 342.1175	Sucrose	0.697–0.735	2.049–2.062	CB1, CB2, CB5, CB7
C_2_H_7_NO_3_S	126.0223 125.0151	Taurine	0.708–0.713	2.089–2.096	CB3, CB5, CB6, CB7, CB8
C_6_H_12_O_6_	203.0531 180.0638	Mono-saccharide	0.704–0.727	2.132–2.150	CB1, CB2, CB5, CB6, CB7
C_6_H_6_N_2_O	123.0555 122.0483	Niacinamide	0.743–0.745	2.202–2.203	CB3, CB4, CB5, CB6, CB7, CB8
C_10_H_16_N_2_O_8_	293.0988 292.0914	EDTA	0.742–0.785	2.220–2.227	All
C_10_H_16_N_2_O_8_	315.0807 292.0914	EDTA	0.761–0.825	2.271–2.287	All
C_6_H_8_N_2_O_7_	215.0170 192.0277	Citric acid	0.779–0.780	2.332–2.334	CB3, CB4, CB5, CB6, CB7, CB8
C_7_H_7_NO_2_	138.0665 137.0477	Trigonelline	1.072–1.078	3.266–3.281	All
C_8_H_10_N_4_O_2_	195.0882 194.0809	Caffeine	1.078–1.080	3.273–3.275	All
C_12_H_19_C_l3_O_8_	419.0048 396.0157	Sucralose	1.145–1.155	3.494–3.497	CB3, CB4, CB7, CB8

## Conclusion

The application of a new commercially available column end fitting (the RFS end fitting) has been demonstrated in this work, specifically for the high-throughput analysis of energy drinks. Notable benefits for the use of the RFS end fitting in this type of assay include:

1. Increased throughput—the increase in column performance and decrease in pressure allowed for faster operation, facilitating run times that were less than one-third of conventional operation, without loss of separation performance.
2. Better column performance—the use of the RFS end fitting was noted to improve peak shape, owing to the RFS editing radially segmenting the column flow, such that only the most homogeneous portion of the elution band (the radial central portion) was detected.
3. MS cleanliness—with one-third of the sample analysis in RFS mode being passed through the MS, less contamination of the MS would be expected, and this could translate to less downtime and general MS interferences.

However, there are still some disadvantages to using the RFS end fitting, namely the loss of sensitivity—peak heights were notably decreased when the column was operated in RFS mode, owing to less sample mass being sent to the detector. However, this is an inherent limitation of any flow splitter, and it is important to note that while traditional flow splitters share this limitation, the RFS design is the only known flow splitter to increase performance and throughput, justifying the decreased sensitivity.

The analysis of caffeine in a range of beverages showed that the concentrations matched the specification, but it was also seen that a co-eluting component, trigonelline, could be a problematic impurity under these conditions if using a non-specific detector.

## Data Availability

Data is available upon request to the authors.
